# Integrating CAR T-Cell Therapy and Transplantation: Comparisons of Safety and Long-Term Efficacy of Allogeneic Hematopoietic Stem Cell Transplantation After CAR T-Cell or Chemotherapy-Based Complete Remission in B-Cell Acute Lymphoblastic Leukemia

**DOI:** 10.3389/fimmu.2021.605766

**Published:** 2021-05-07

**Authors:** Yan-Li Zhao, De-Yan Liu, Rui-Juan Sun, Jian-Ping Zhang, Jia-Rui Zhou, Zhi-Jie Wei, Min Xiong, Xing-Yu Cao, Yue Lu, Jun-fang Yang, Xian Zhang, Dao-Pei Lu, Peihua Lu

**Affiliations:** ^1^ Department of Bone Marrow Transplantation, Hebei Yanda Lu Daopei Hospital, Langfang, China; ^2^ Department of Hematology and Immunology, Hebei Yanda Lu Daopei Hospital, Langfang, China; ^3^ Lu Daopei Institute of Hematology, Beijing, China

**Keywords:** CD19 CAR T-cell therapy, relapse/refractory B cell acute lymphoblastic leukemia, allogeneic hematopoietic cell transplantation, survival, relapse

## Abstract

Patients often undergo consolidation allogeneic hematopoietic stem cell transplantation (allo-HSCT) to maintain long-term remission following chimeric antigen receptor (CAR) T-cell therapy. Comparisons of safety and efficacy of allo-HSCT following complete remission (CR) achieved by CAR-T therapy *versus* by chemotherapy for B-cell acute lymphoblastic leukemia (B-ALL) has not been reported. We performed a parallel comparison of transplant outcomes in 105 consecutive B-ALL patients who received allo-HSCT after achieving CR with CAR-T therapy (n=27) or with chemotherapy (n=78). The CAR-T-allo-HSCT group had more patients in second CR compared to the chemotherapy-allo-HSCT group (78% *vs.* 37%; p<0.01) and more with complex cytogenetics (44% *vs.* 6%; p<0.001) but the proportion of patients with pre-transplant minimal residual disease (MRD) was similar. The median follow-up time was 49 months (range: 25-54 months). The CAR-T cohort had a higher incidence of Grade II-IV acute graft-*versus*-host disease (aGVHD 48.1% [95% CI: 46.1-50.1%] *vs.* 25.6% [95%CI: 25.2-26.0%]; p=0.016). The incidence of Grade III-IV aGVHD was similar in both groups (11.1% *vs.*11.5%, p=0.945). The overall incidence of chronic GVHD in the CAR-T group was higher compared to the chemotherapy group (73.3% [95%CI: 71.3-75.3%] *vs.* 55.0% [95%CI: 54.2-55.8%], p=0.107), but the rate of extensive chronic GVHD was similar (11.1% *vs.*11.9%, p=0.964). Efficacy measures 4 years following transplant were all similar in the CAR-T *vs.* the chemotherapy groups: cumulative incidences of relapse (CIR; 11.1% vs.12.8%; p=0.84), cumulative incidences of non-relapse mortality (NRM; 18.7% *vs.* 23.1%; p=0.641) leukemia-free survival (LFS; 70.2% *vs.* 64.1%; p=0.63) and overall survival (OS; 70.2% *vs.* 65.4%; p=0.681). We found that pre-transplant MRD-negative CR predicted a lower CIR and a higher LFS compared with MRD-positive CR. In conclusion, our data indicate that, in B-ALL patients, similar clinical safety outcomes could be achieved with either CD19 CAR T-cell therapy followed by allo-HSCT or chemotherapy followed by allo-HSCT. Despite the inclusion of more patients with advanced diseases in the CAR-T group, the 4-year LFS and OS achieved with CAR T-cells followed by allo-HSCT were as remarkable as those achieved with chemotherapy followed by allo-HSCT. Further confirmation of these results requires larger, randomized clinical trials.

## Introduction

Refractory/relapsed (R/R) B-cell acute lymphoblastic leukemia (B-ALL) is a leading cause of morbidity and mortality in children and young adults (1–3). Allogeneic hematopoietic stem cell transplantation (allo-HSCT) is often undertaken for high-risk R/R B-ALL patients. However, many R/R patients are never able to achieve a complete remission (CR) following chemotherapy and are not referred for allo-HSCT. Therefore, relapse rates among these patients remain high despite the potential cure that is possible for B-ALL patients with an allo-HSCT. Achieving a CR prior to allo-HSCT has been shown to improve outcomes for these R/R B-ALL patients including improving leukemia free survival (LFS) following transplantation (4, 5).

In recent years, clinical trials with anti-CD19+ chimeric antigen receptor T-cell (CAR-T) therapy have demonstrated high CR rates of ~70% to 90% in patients with R/R B-ALL (6–12) and offer the hope of a potential cure for those patients who are otherwise refractory or relapsed following chemotherapy. However, remission following CAR-T therapy is often not durable with about half of CR patients relapsing within 1 year of therapy (6, 10, 13, 14). There is accumulating evidence from recent studies demonstrating that CAR-T therapy followed by allo-HSCT could potentially result in higher rates of durable, long-term remission for pediatric R/R B-ALL and reduce the relapse rates seen with CAR T-cell therapy alone (6, 8, 15–17). Yet there is still controversy around the safety and efficacy of allo-HSCT following CAR T-cell therapy and the ability to achieve long-term LFS with this sequential, dual immunotherapy. Some studies have reported high relapse rates and higher treatment related mortality following transplant after CAR-T therapy, resulting in no improvement of LFS and overall survival (OS) when compared to CAR T-cell therapy alone (11, 18).

In the present study, we conducted a parallel comparison of outcomes among R/R B-ALL patients who achieved remission from either CAR T-cell therapy or chemotherapy and who subsequently underwent allo-HSCT. We report safety and efficacy results in these two cohorts.

## Materials and Methods

### Patients

**Figure 1:**
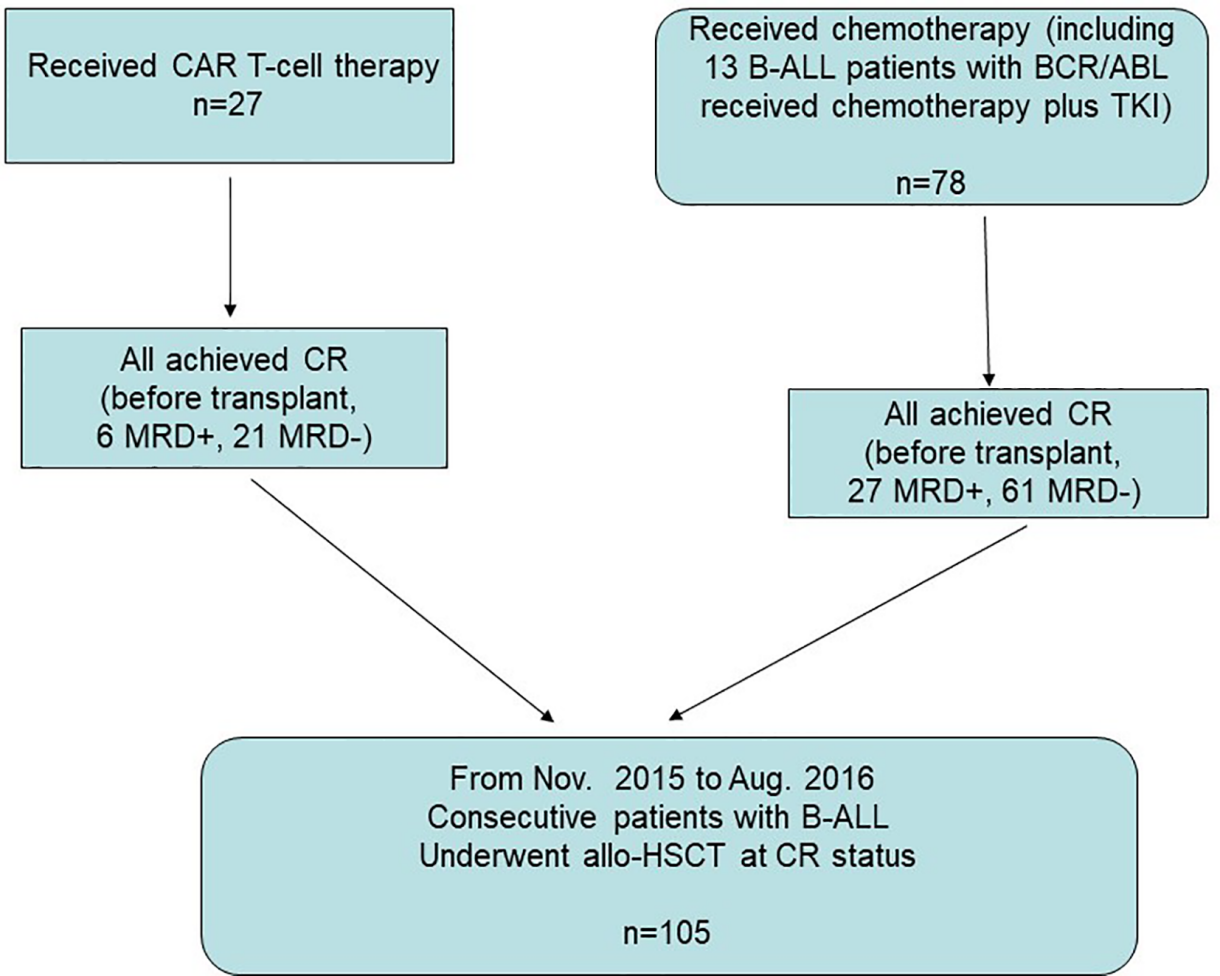
Enrollment, and parallel comparison. Between November 2015 and August 2016, 105 consecutive B-ALL patients who underwent allo-HSCT after achieving CR either from CAR-T therapy (n=27) or chemotherapy (n=78) were enrolled for parallel comparison.

We included 105 consecutive B-ALL patients who underwent allo-HSCT after achieving CR either from CAR-T therapy (n=27) or chemotherapy (n=78) at the Hebei Yanda Lu Daopei Hospital between November 2015 and August 2016. Details on the enrollment of the CAR-T group and chemotherapy group (including 13 B-ALL patients with *BCR/ABL* who received chemotherapy plus a tyrosine kinase inhibitor) are shown in **Figure 1**.

The study protocol was approved by the ethics committee of the Hebei Yanda Lu Daopei Hospital. Informed consent was obtained from the patients according to the Declaration of Helsinki.

### CD19+ CAR T-Cell Therapy and CAR-T-Related Side Effects

CD19+ CAR T-cell therapy was performed according to previously described methods (6). Briefly, we used a second generation CD19+ lentiviral vector that also expressed the co-stimulatory 4-1BB molecule (CAR-T clinical trials No: ChiCTR-IIh-16008711). Before CAR T-cell infusion, patients received lymphodepleting chemotherapy consisting of fludarabine (30 mg/m^2^/day) and cyclophosphamide (250 mg/m^2^/day) (FC) on days -5, -4, and -3. Cytokine release syndrome (CRS) and neurotoxicity grading were performed according to previously described methods (19–22).

### Clinical Transplant Protocol

Before transplantation, patients received intensive myeloablative conditioning regimens. Total body irradiation (TBI) plus cyclophosphamide/fludarabine-based chemotherapy or busulfan (Bu) plus cyclophosphamide/fludarabine-based were used according to each patient’s status. TBI-based regimes were preferred if no contraindications such as severe pulmonary complications were observed. TBI was given using a horizontal beam in a linear accelerator. Patients in the TBI group received conditioning with fractioned TBI (200cGy Bid for 5-6 doses). Patients in the Bu group received Bu (0.8mg/kg i.v. Q6h for 16 doses) on days -8 to -6. TBI or Bu was followed by cyclophosphamide 1.8 g/m^2^/day for 2 days or fludarabine 30mg/m^2^/day for 5 days. Rabbit anti-T Cell Globulin (ATG, Fresenius; totally 20mg/kg divided by 4 days) or thymoglobuline (ATG, Sanofi-Aventis, total dose of 7.5mg/kg divided by 4 days) were used on days -5 to -2 in mismatched unrelated transplants and haploidentical transplants (Haplo-HSCT). Cyclosporine, short-term methotrexate (15 mg/m^2^ on day +1, then 10 mg/m^2^ on days +3, +6, and +11 intravenously after transplantation), and mycophenolate mofetil were used for graft-versus-host disease (GVHD) prophylaxis. Grafts were granulocyte colony-stimulating factor (G-CSF) mobilized bone marrow (BM) and peripheral blood (PB) cells as described previously (23).

Acute GVHD (aGVHD) was diagnosed and graded according to modified Glucksberg criteria (24–26). Chronic GVHD (cGVHD) was evaluated using National Institutes of Health consensus criteria (27, 28) and aGVHD treatment was described previously (23, 24). Thrombotic microangiopathy (TMA) was diagnosed according to the Jodele criteria (29).

### Analysis of Chimerism

Hematopoietic chimerism was evaluated by PCR amplification of short tandem repeats (STR) using both bone marrow and CD3+ cells from PB samples collected at 1, 2, 3, 6, 9, and 12 months after transplant and at 6-month intervals thereafter. Complete donor chimerism was defined as the presence of ≥95% of the donor-type.

### Statistics

Comparisons of patient characteristics between the two groups were performed using the Mann-Whitney U-test for continuous variables and χ2 for categorical data. The probabilities of survival were calculated using the Kaplan-Meier method. Cumulative incidences were estimated for aGVHD, non-relapse mortality (NRM), and relapse to accommodate for competing risks. Death and relapse were competing events for aGVHD and death was a competing event for cGVHD. NRM was the competing event for relapse and vice versa. Hazard ratios (HRs) for clinical outcomes were estimated in a multivariate analysis using Cox proportional hazards regression with a backward stepwise model selection approach. The following variables were included: gender, patient age (<14 years *vs.* ≥14 years), pre-HSCT treatment (CAR-T *vs.* chemotherapy), disease status pre-transplant (≥CR2 *vs.* CR1), MRD status pre-transplant (positive *vs.* negative), poor risk chromosomes (yes *vs.* no), conditioning regimens (TBI-based *vs.* Bu-based), donor type (alternative donor *vs.* identical unrelated or sibling donor), donor-recipient gender matching (female to male *vs.* others), course from diagnosis to transplant, and mononuclear, CD3+ and CD34+ cell counts (using the median value as the cut-off point). Independent variables with P > 0.1 were sequentially excluded from the model, and P < 0.05 was considered to be statistically significant. P values were 2-sided. The SPSS 16 (SPSS Inc./IBM, Armonk, NY, USA) and the R software package (version 4.0.0; http://www.r-project.org) were used for data analyses. Surviving patients were censored on April 30th, 2020.

### Definitions

CR and CR with incomplete count recovery (CRi) were defined in accordance with the National Comprehensive Cancer Network (NCCN) guideline, version 1.2018 (30). Minimal residual disease (MRD)-negative status was defined as the absence of leukemia cells in BM determined by multiparameter flow cytometry (FCM, sensitivity, 1:10,000), and the absence of leukemia-associated fusion gene in BM determined by real-time quantitative PCR (RT-PCR). Hypodiploidy, complex karyotype, t(v;11q23) or t(9;22) (q34;q11.2) determined by G band or FISH were defined as poor risk chromosomes according to the NCCN guideline, version 1.2018 (30). LFS and OS were calculated from the date of allo-HSCT to the date of relapse or death or the last follow-up time. The cumulative incidence of relapse (CIR) was calculated from date of allo-HSCT to the date of relapse.

## Results

### Patient Characteristics

The detailed characteristics of the two groups are summarized in **Table 1**. Patients’ median age was 13 years (range: 2–52 years) with a 58/42 male/female ratio. Fifty-three percent of the patients were pediatric (age <14 years) and 47% of patients were adults (age ≥14 years). High WBC counts were observed in 31 patients (30%) at initial diagnosis.

**Table 1:** Patient characteristics.

Characteristics	Total	CAR-T group	Chemotherapy group	P value
No.	105	27	78	
Median age, years (range)	13.0(2-52)	11(3-44)	14.5(2-52)	0.441
Age group, no. (%)				0.236
≥14	49(47)	10(36)	39(50)	
<14	56(53)	17(63)	39(50)	
Median donor age, years (range)	33(8-63)	31(16-54)	33.5(8-63)	0.550
Gender, male, no. (%)	61(58%)	18(67)	43(55)	0.295
With extramedullary disease (EMD), no (%)	15(14)	4(15)	11(14)	0.927
Median duration from diagnosis to HSCT months (range)	13.5(4-123)	20(4-54)	11(4-123)	0.232
Disease risk				0.042
R/R B-ALL, no (%)	70(67)	22(82)	48(62)	
From diagnosis to first relapse time, no. (%)				
<18 months	19(58)	11(41)	8(17)	
18~36 months	19(44)	8(30)	11(14)	
>36 months	16(19)	2(1)	14(18)	
Primary refractory	16(19)	1(0)	15(19)	
Persistent or relapsed MRD	17(16)	5(19)	12(15)	
Others	18(17)	0	18(17)	
Disease status pre-transplant, no. (%)				<0.001
CR1	55(52)	6(22)	49(63)	
≥CR2	50(48)	21(78)	29(37)	
Donor source, no. (%)				0.588
Haplo-d	66(63)	16(59)	50(64)	
MUD	24(23)	8(30)	16(21)	
MSD	15(14)	3(11)	12(15)	
FCM MRD-positive pre-conditioning, no. (%)	33(31)	6(22)	27(35)	0.232
Fusion genes				0.023
BCR-ABL1	13(12)	0	13(17)	
TEL-AML1	9(9)	2(7)	7(9)	
E2A-PBX1	4(4)	3(11)	1(1)	
MLL-AF4	4(4)	0	4(5)	
MLL-AF1P	1(1)	1(4)	0	
MLL-ENL	1(1)	0	1(1)	
Gene mutations				
NRAS/KRAS	12(11)	5(11)	7(9)	0.16
IKZF	9(9)	2(7)	7(9)	0.58
TP53	5(5)	2(7)	3(4)	0.383
Flt3 ITD/KTD	4(4)	1(4)	3(4)	0.728
High risk cytogenetics, no. (%)	48(46)	16(59)	32(41)	0.103
Complex cytogenetic, no (%)	17(16)	12(44)	5(6)	<0.001
Donor-recipient gender match, n (%)				0.263
Female to male	19(18)	7(26)	12(15)	
Others	86(82)	20(74)	66(85)	
Conditioning regimens, no. (%)				0.335
TBI-based	87(83)	24(89)	63(81)	
Bu-based	18(17)	3(11)	15(19)	
Median CD34 cells,×10^6^/kg(range)	4.45(1.76-12.23)	4.6(1.76-10.18)	4.41(2.02-12.23)	0.428
Median CD3 cells,×10^8^/kg(range)	1.66(0.44-4.99)	1.83(0.85-3.04)	1.59(0.44-4.99)	0.491
Graft type, no. (%)				0.41
BM+PB	80(76)	19(70)	61(78)	
PB	25(24)	8(30)	17(22)	
Neutrophil engraftment, days (range)	14(10-29)	14(11-20)	4(10-29)	0.973
Platelet engraftment, days (range)	12(4-47)	14(5-47)	12(4-32)	0.026
Median follow-up time in survivor, months (range)	49 (25-54)	49 (44-53)	49 (25-54)	0.831

The median time from CAR T-cell therapy to HSCT was 84 days (range: 35-293 days). CRS was observed in the majority of the patients in the CAR T-cell therapy group. Grade 1 (56%) and Grade 2 (26%) CRS made up the majority of CRS cases. Severe CRS occurred in 15% of patients—11% of patients had Grade 3, and 4% of patients had Grade 4 CRS. A total of four patients had Grade 3 neurotoxicity with seizures.

In the CAR-T group, 22 (81%) patients had R/R B-ALL, and 5 (19%) had persistent or relapsed MRD after hematological remission. Among the 21 relapsed patients in the CAR T-cell group, the median time from diagnosis to first relapse was 17 months (range: 3-47 months). Following relapse, 17 patients failed to regain CR after a median 2 courses of chemotherapy (range: 1-5 courses) and afterwards underwent CAR T-cell therapy. Four patients who had relapsed during consolidation chemotherapy received CAR-T therapy directly. In the chemotherapy group, 48 (62%) had R/R B-ALL, and 12 (15%) had persistent or recurrent MRD. The median time from diagnosis to last relapse of the 33 relapsed patients was 31 months (range: 2-120 months). One patient relapsed 3 times within 10 years. Among 18 (17%) patients in the chemotherapy group, 9 presented with high risk ALL. As shown in **Table 1**, in the CAR-T group, prior to allo-HSCT, 22% (6/27) of the patients were in CR1 compared to 63% (49/78) of the patients in CR1 in the chemotherapy group. Compared to the chemotherapy group, the CAR-T group had more patients who were ≥CR 2 (78% *vs.* 37%, respectively; p<0.001).

As assayed by FCM and RT-PCR, 22% of patients in the CAR-T group and 35% of patients in the chemotherapy group had MRD detected pre-transplant (p=0.232). The proportion of patients with extramedullary diseases at diagnosis and at relapse before transplant were not significantly different between the CAR-T group and the chemotherapy group (p=0.927). There was no significant difference in the median time from diagnosis to transplant (13.5 months [range: 4-123 months]). Complex chromosomes were present in 44% of patients in the CAR-T group and 12% of the chemotherapy group (p<0.001). There was significant difference in the presence of fusion genes (p=0.023). Poor risk BCR-ABL1 (n=13) and MLL-AF4 (n=4) were exclusively observed in the chemotherapy group (**Table 1**).

### Donor Source, Graft, Conditioning Regimens and Engraftment

In the CAR-T group, 59% of patients received a transplant from haploidentical donors (Haplo-D), 30% from matched unrelated donors (MUD), and 11% from HLA-matched sibling donors (MSD). In the chemotherapy group, 64% of patients received a transplant from Haplo-Ds, 21% from MUDs and the remaining 15% of patients received a transplant from MSDs. There were no significant differences among different donor types, the donors’ age and gender between the CAR-T and chemotherapy groups. In addition, there were no differences in the median mononuclear cells, CD34 and CD3 between the two groups (**Table 1**).

Myeloablative conditioning regimens were administered with TBI cyclophosphamide/TBI-fludarabine in 83% of patients and Bu cyclophosphamide/fludarabine in 17% of patients. There was no significant difference in conditioning regimens observed between the groups.

All patients achieved sustained neutrophil engraftment after a median of 14 days (range: 11-20 days) in the CAR-T group and 14 days (range: 10-29 days) in the chemotherapy group (p=0.97). Platelet engraftment failure occurred in 2 patients (7%) in the CAR-T group. One patient died of severe acute GVHD on day 27 after transplantation, and one died of infection at 68 days post- transplantation. All the 78 patients in the chemotherapy group achieved sustained platelet engraftment. There was a significant difference in platelet engraftment between the two groups (p=0.026). Post-transplant, the median day of platelet engraftment was significantly longer in the CAR-T group (14 days, range: 5-47 days) compared to the chemotherapy group (12 days, range: 4-32 days) (p=0.026).

No graft failure occurred (except that one patient had poor graft function) and rapid achievement of full donor chimerism was confirmed in all patients by day 30. No significant difference between the two groups was observed.

### Incidence of GVHD

**Figure 2:**
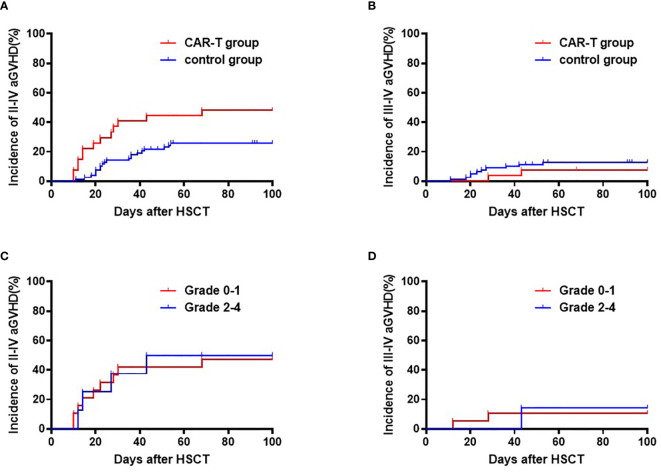
Cumulative incidences of Grade II-IV and Grade III-IV acute GVHD. **(A)** Cumulative incidences of Grade II-IV acute GVHD: CAR-T group: 48.1% (95% CI:46.1, 50.1%) *vs.* chemotherapy group: 25.6% (95% CI: 25.2, 26.0%); p=0.016. **(B)** Cumulative incidences of Grade III-IV acute GVHD: CAR T-cell group: 11.1% (95% CI: 10.3, 11.9%) *vs.* chemotherapy group: 11.5% (95% CI: 11.3, 11.7%); p=0.945. **(C)** Cumulative incidences of Grade II-IV acute GVHD: CRS Grade 0-I 47.4% (95% CI: 44.7, 50.1%) *vs.* CRS Grade II-IV: 50.0% (95% CI:42.6, 57.4%); p=0.95. **(D)** Cumulative incidences of Grade III-IV aGVHD: CRS Grade 0-I: 10.5% (95% CI: 9.5, 11.5%) *vs.* CRS Grade II-IV: 12.5% (95% CI: 9.4, 15.6%); p=0.92.

The cumulative incidence of Grade II-IV aGVHD was higher in the CAR T-cell group compared to the chemotherapy group (48.1% [95% CI: 46.1, 50.1%] *vs.* 25.6% [95% CI: 25.2, 26.0%], respectively; p=0.016), while the incidence of Grade III-IV aGVHD were similar between the two groups (11.1% [95% CI: 10.3, 11.9%] *vs.* 11.5% [95% CI: 11.3, 11.7%], respectively; p=0.945) (**Figures 2A, B**). A low versus high grade CRS (Grade 0-1 *vs.* Grade 2-4) before transplant did not have significant effects on Grade II-IV aGVHD (47.4% [95% CI: 44.7, 50.1%] *vs.* 50.0% [95% CI: 42.6, 57.4%] among the CAR-T and chemotherapy groups, respectively; p=0.95) or on Grade III-IV aGVHD (10.5% [95% CI: 9.5, 11.5%] *vs.* 12.5% [95% CI: 9.4, 15.6%]; p=0.92) after transplant (**Figures 2C, D**).

**Figure 3:**
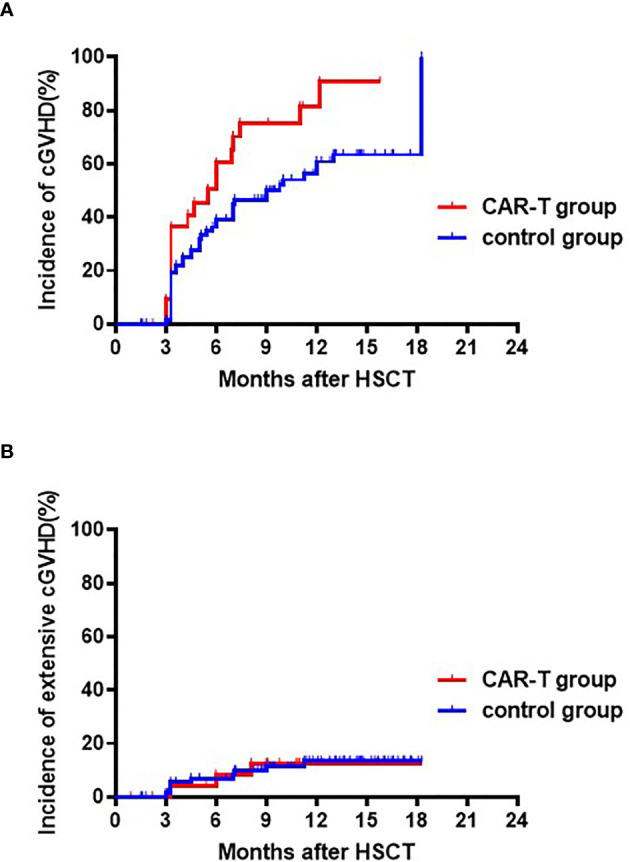
Cumulative incidences of chronic and extensive chronic GVHD. **(A)** Cumulative incidences of chronic GVHD: CAR T-cell group: 73.3% (95% CI: 71.3, 75.3%) *vs.* chemotherapy group: 55.0% (95% CI: 54.2, 55.8%); p=0.107. **(B)** Cumulative incidences of extensive chronic GVHD: CAR T-cell group: 11.1% (95% CI:10.3, 11.9%) *vs.* the chemotherapy group: 11.9% (95% CI: 11.7, 12.1%); p=0.964.

For patients surviving over 100 days after transplantation, cumulative incidence of cGVHD at 18 months were higher in the CAR-T group, but this difference did not reach statistical difference (71.3% [95% CI: 71.3, 75.3%] *vs.* 55.0% [95% CI: 54.2, 55.8%], p=0.107) (**Figure 3A**). Cumulative incidence of extensive cGVHD at 18 months was similar between the CAR-T and chemotherapy groups (11.1% [95% CI: 10.3, 11.9%] *vs.* 11.9% [95% CI: 11.7, 12.1%]; p=0.964) (**Figure 3B**).

### CIR After HSCT

**Figure 4:**
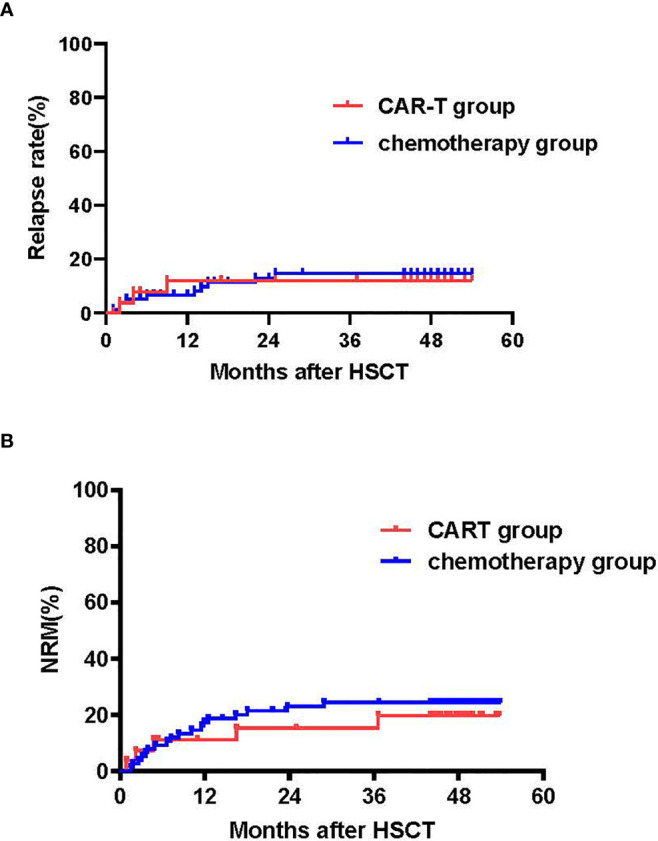
Cumulative incidences of relapse (CIR) and NRM. **(A)** Cumulative incidence of relapse: CAR T-cell group: 4-year CIR of 11.1% (95% CI: 10.3, 11.9%) *vs.* chemotherapy group: 12.8% (95% CI:12.6, 13.0%) (p=0.84). **(B)** Cumulative incidence of NRM: CAR T-cell group: 4-year NRM of 18.7% (95% CI:17.5, 19.9%) *vs.* chemotherapy group: 4-year NRM of 23.1% (95% CI:22.7, 23.5%); p=0.64.

The CIRs at 4 years following transplant were 11.1% [95%CI: 10.3,11.9%] for the CAR-T group versus 12.8% [95% CI: 12.0-13.6%] for the chemotherapy group (p=0.84) (**Figure 4A**). Univariate analysis showed that disease status (HR 3.87, [95% CI 1.09-13.7], p=0.027) and MRD before transplantation (HR 2.81 [95%CI 0.961-8.24], p=0.056) were predictive factors for relapse. The multivariate analysis confirmed these predictive effects of relapse (HR 4.10, [95% CI1.13-14.84], p=0.031 and HR 3.02, [95% CI1.02-8.96], p=0.046, for disease status and MRD before transplant, respectively) (**Table 2**).

**Table 2:** Univariate and multivariate analysis of risk factors for clinical outcomes.

	II-IV GVHD						III-IV GVHD						CIR						NRM						LFS					
	Univariate			Multivariate			Univariate			Multivariate			Univariate			Multivariate			Univariate			Multivariate			Univariate			Multivariate		
	HR	95% Cl	p value	HR	95% Cl	p value	HR	95% Cl	p value	HR	95% Cl	p value	HR	95% Cl	p value	HR	95% Cl	p value	HR	65% Cl	p value	HR	65% Cl	p value	HR	65% Cl	p value	HR	65% Cl	p value
Age(≥14 *vs.* <14yrs)	0.785	0.395-1.56	0.49				0.339	0.0921-1.25	0.088	0.506	0.1081-2.37	0.39	1.23	0.418-3.62	0.708				2.09	0.89-4.91	0.084				1.8	0.921-3.51	0.079	1.652	0.84-3.25	0.15
Treatment pre-HSCT (CAR-T vs. control)	2.36	1.18-4.75	0.016				0.956	0.263-3.47	0.945				0.873	0.241-3.17	0.836				0.791	0.295-2.12	0.641				0.827	0.38-1.8	0.631			
Disease status(≥CR2 *vs.* CR 1)	1.09	0.557-2.15	0.769				0.494	0.152-1.6	0.239				3.87	1.09-13.7	0.027	4.1	1.13-14.84	0.031	0.774	0.344-1.74	0.54				1.54	0.808-2.93	0.192			
MRD(pos *vs.* neg)	1.09	0.536-2.23	0.81				2.29	0.75-7.02	0.142				2.81	0.961-8.24	0.056	3.02	1.02-8.96	0.046	1.45	0.638-3.32	0.38				2.21	1.16-4.24	0.017	2.105	1.09-4.06	0.027
Poor risk chromosomes (yes vs. no)	1.5	0.76-2.95	0.245				2.48	0.735-8.17	0.125				0.709	0.236-2.13	0.543				0.622	0.263-1.47	0.271				0.614	0.313-1.21	0.151			
Conditioning(TBI-based *vs.* Bu-based)	0.888	0.365-2.16	0.795				0.254	0.0815-8.17	0.015	0.387	0.0929-1.61	0.19	0.655	0.181-2.38	0.523				0.937	0.314-2.79	0.903				0.883	0.384-2.03	0.765			
Donor type (haplo *vs.* MUD/MSD)	0.894	0.45-1.78	0.753				3.17	0.715-14	0.113				1.33	0.413-4.31	0.629				0.917	0.402-2.09	0.84				1.25	0.646-2.42	0.516			
donor-recipients gender matched(female to male *vs.* others)	1.16	0.464-2.89	0.761				3.37	1.07-10.6	0.0375	1.885	0.4163-8.54	0.41	1.15	0.271-4.89	0.853				0.586	0.142-2.42	0.457				0.61	0.234-1.59	0.33			
course from diagnosis to transplant (≥ median vs. <median)	0.883	0.45-1.73	0.719				0.455	0.141-1.47	0.188				2.34	0.732-7.48	0.144				0.6	0.262-1.37	0.225				1.05	0.552-1.98	0.893			
MNC (≥median *vs.* <median)	0.59	0.303-1.15	0.3				0.996	0.351-2.82	0.934				1.11	0.412-3	0.886				0.926	0.433-1.98	0.871				1.12	0.62-2.01	0.616			
CD3 (≥median *vs.* <median)	1.17	0.596-2.29	0.655				0.713	0.228-2.23	0.561				0.421	0.13-1.36	0.137				1.13	0.506-2.53	0.766				0.824	0.432-1.57	0.559			
CD34 (≥median *vs.* <median)	1.01	0.511-1.98	0.986				0.682	0.219-2.12	0.51				0.413	0.129-1.32	0.128				0.916	0.408-2.06	0.834				0.576	0.303-1.1	0.096	0.644	0.34-1.22	0.18
cGVHD (limited *vs.* extensive vs.																														

A total of 13 patients relapsed after transplant, three in CAR-T group and 10 in the chemotherapy group. All except one patient died at a median time of day 283 (range: 48-1116) after transplant. The patient that survived relapsed following a second haploidentical transplant underwent donor CD19 CAR T-cell therapy and remains in remission.

In the CAR T-cell group, 6 patients were MRD positive (MRD+CR) before transplant, including 5 patients who were CD19 negative (CD19-) and MRD+CR. Two of the CD19- MRD+CR patients relapsed with CD19- leukemia at Day 60 and at Day 275, and consequently died at Day 270 and Day 336 after transplant, respectively. Another patient died of severe GVHD on Day 27. The remaining three patients survived in remission at Month 46, 47, and 49, respectively.

### Infection, TMA and NRM

No remarkable differences were observed in cytomegalovirus (CMV) reactivation (52% *vs.* 50%, p=0.93) between the CAR T-cell and chemotherapy groups, respectively. There were also no differences in rates of transplant-associated TMA (TA-TMA) between the CAR T-cell and chemotherapy groups, respectively (15% *vs.* 14%, p=0.51). In the chemotherapy group, three patients were diagnosed with viral pneumonia and died. Incidences or non-relapse mortality (NRM) within 100 days were 7.4% (95% CI:6.8-8.0%) and 5.1% (95% CI: 4.9-5.3%) (p=0.64). The NRM at 1 and 4 years after transplantation was 11.1% (95% CI: 10.3-11.9%) and 18.7% (95% CI: 17.5, 19.9%), respectively, for the CAR-T group versus 16.7% (95% CI [16.3-17.1%] and 23.1% (95% CI: 22.7, 23.5%) for the chemotherapy group (p=0.64) (**Figure 4B**).

### LFS, OS and Cause of Mortality

**Figure 5:**
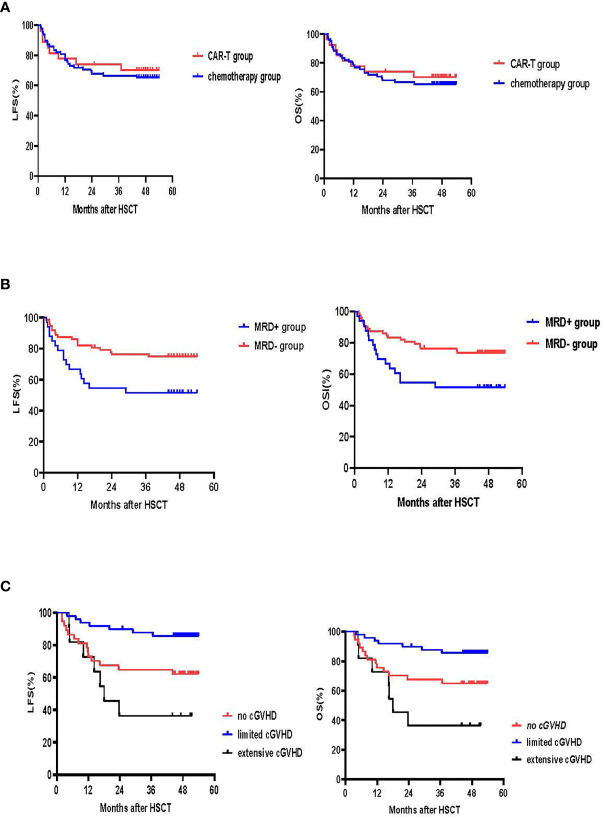
LFS and OS. **(A)** LFS and OS in the CAR-T and chemotherapy groups. The 4-year LFS for the CAR-T group was 70.2% (95% CI:53.0, 87.4%) *vs.* 64.1% (95% CI:53.5, 74.7%) for the chemotherapy group (p=0.63). The 4-year OS for the CAR-T group was 70.2% (95% CI:53.0, 87.4%) *vs.* 65.4% (95% CI:54.8, 76.0%) for the chemotherapy group (p=0.681) **(B)** LFS and OS according to MRD. The 4-year LFS for patients who achieved MRD- CR was 72.2% (95% CI:61.8, 82.6%) and 51.5% (95% CI:34.4, 68.6%) for those that had an MRD+ CR (p=0.024). The 4-year OS for the MRD- CR group was 73.6% (95% CI:63.4, 83.8%) and 51.5% (95% CI:34.4, 68.6%) for the MRD+CR group (p=0.02). **(C)** LFS and OS according to cGVHD. 4-year LFS for patients without cGVHD was 62.2% (95% CI:46.5, 77.9%), 85.6% (95% CI:75.8,95.4%) for those with limited cGVHD and 36.4% (95% CI:8.0, 64.8%) for those with extensive cGVHD (no *vs.* limited cGVHD, p=0.009; limited *vs.* extensive cGVHD, p<0.001; no *vs.* extensive cGVHD, p=0.20). 4-year OS for the no cGVHD group was 64.9% (95% CI:49.6, 80.2%), 85.6% for the limited cGVHD group (95% CI:75.8, 95.4%) and 36.4% for the extensive cGVHD group (95% CI:8.0, 64.8%) (no *vs.* limited cGVHD, p=0.019; limited *vs.* extensive cGVHD, p<0.001; no *vs.* extensive cGVHD, p=0.123).

With a median follow-up of 49 months (range: 44-54 months) for surviving patients, LFS and OS at 4 years were similar in the CAR-T and chemotherapy groups (LFS: 70.2% [95% CI: 53.0, 87.4%] *vs.* 64.1% [95% CI: 53.5, 74.7%], p=0.63; OS: 70.2% [95% CI: 53.0, 87.4%] *vs.* 65.4% [95%CI:54.8, 76.0%], p=0.681) (**Figure 5A**).

Univariate and multivariate analysis showed that MRD prior to transplant was a negative prognostic factor for LFS (p=0.024 for univariate-analysis and p=0.016 (HR 2.6 [95% CI: 1.2, 5.8] for multivariate-analysis). The 4-year LFS for the MRD-CR and MRD+CR groups was 72.2% [95% CI: 61.8, 82.6%] and 51.5% [95% CI: 34.4, 68.6%], respectively (p=0.024). The 4-year OS for MRD-CR patients was 73.6% (95%CI: 63.4, 83.8%) and MRD+CR of 51.5% (95%CI: 34.4, 68.6%) (p=0.02), respectively (**Figure 5B**).

For the 97 patients who survived over 100 days after transplant, 4-year LFS for no cGVHD, and limited and extensive cGVHD was 62.2% (95% CI: 46.5, 77.9%), 85.6% (95% CI: 75.8, 95.4%) and 36.4% (95% CI: 8.0, 64.8%), respectively (no *vs.* limited cGVHD, p=0.009; limited *vs.* extensive cGVHD, p<0.001; no *vs.* extensive cGVHD, p=0.20). The 4-year OS for the no cGVHD cohort was 64.9% (95% CI:49.6, 80.2%), 85.6% (95% CI: 75.8, 95.4%) for the limited cGVHD cohort and 36.4% (95% CI: 8.0, 64.8%) for the extensive cGVHD cohort (no *vs.* limited cGVHD, p=0.019; limited *vs.* extensive cGVHD, p<0.001; no *vs.* extensive cGVHD, p=0.123) (**Figure 5C**).

At the time of the latest follow up in April 2020, 29 patients had died. The primary causes of death were relapse (8 patients), GVHD (8 patients), infection (5 patients) and TMA (5 patients) (**Table 3**).

**Table 3:** Causes of death in the CAR-T and chemotherapy groups.

Causes	Total	CAR-T group	Chemotherapy group
All causes of death	34	8	26
Relapse	11	3	8
NRM	23	4	18
GVHD	8	1	7
Infection	6	2	4
TMA	5	1	4
Graft failure or rejection	1		1
Malignant arrhythmia	1		1
Acute pancreatitis	1	1	
Organic pneumonia	1		1

## Discussion

Recently CAR-T therapy has shown dramatic initial responses with CR rates approaching 80-90% among R/R B-ALL patients (6–12). However, risk of relapse remains a major problem for these patients. Allo-HSCT after CAR-T therapy may have a consolidative role to further improve the durability of remission for these patients. However, whether prior CAR-T therapy can potentially increase the transplant-related mortality and toxicity remain a concern. In the present study, we compared the safety and efficacy of allo-HSCT in patients in patients after achieving CR either post CAR-T or after chemotherapy with a median follow-up of 4 years. To our knowledge, this is the first analysis comparing B-ALL patient outcomes after allo-HSCT following either prior CAR T-cell therapy or chemotherapy.

Although this is not a randomized trial, our parallel cohort study showed a similarly high LFS (70.2% *vs.* 64.1%) and OS (70.2% *vs.* 65.4%) after a median follow-up of 4 years in patients who received allo-HSCT after achieving CR from CAR-T therapy (n=27) or after achieving CR following chemotherapy (n=78), even despite having significantly more patients with advanced disease and refractory/relapsed status in the CAR-T group. There was no graft-failure in either group. The incidences of NRM, TMA and CMV reactivation within both groups were similar.

Hematopoietic reconstitution is one of the key issues in heavily pre-treated B-ALL patients after allo-HSCT. In our study, all patients achieved prompt and sustained neutrophil engraftment, at a median 14 days and achieved 100% donor chimerism in bone marrow and blood on day 28 in both the CAR-T and chemotherapy groups. Nevertheless, the engraftment of platelets was significantly slower in the CAR-T group compared to the chemotherapy group (Day 14 *vs.* Day 12, p=0.026). One possible reason may be due to the higher incidence of aGVHD and corresponding glucocorticoids treatment of patients in the CAR-T group. In addition, cytokine storm subsequent to CAR-T therapy might impair the endothelium system in transplant recipients (6–9), including the hematological microenvironment.

GVHD remains a major cause of morbidity and mortality following allo-HSCT. The reports of incidence and severity of GVHD after transplant post CAR T-cell therapy have been very limited. In a study by Shadman et al. from the University of Washington, it reported incidence of Grade II-IV and Grade III-IV acute GVHD of 69% and 25%, respectively (17). Jiang et al. reported no severe aGVHD except for mild skin rash and diarrhea (Grade ≤2) among 21 patients (31). In our study, Grade II-IV and Grade III-IV aGVHD were 48% and 11%, respectively, in the CAR-T group. Further analysis showed that the grade of CRS had no influence on the incidence and severity of aGVHD. Considering the limited size of the CAR-T group in our study, further clinical trials are necessary to verify the effect of CRS on aGVHD after transplantation. We found higher incidence of Grade II-IV aGVHD in the CAR T-cell group, but similar incidence of severe aGVHD compared to the chemotherapy group. Regarding the cGVHD, the overall incidence of cGVHD in the CAR-T group was higher, but the rate of extensive cGVHD (11.1% *vs.* 11.9%, in the CAR T-cell and chemotherapy groups, respectively p=0.96) was similar between the groups. There was a relative higher incidence of cGVHD in our study. It is likely because the major donor type was haplo donor (67%) in our study. In addition, considering the high risk of recurrence in this group of patients with advanced disease, immunosuppressants were withdrawn as soon as possible. And in some very high-risk patients, prophylactic DLI and interferon were applied to gain a limited chronic GVHD status in both groups. The incidence of the cGVHD was similar to our previous reports of haplo-HSCT with ATG (23, 32).

Neurotoxicity is a relatively common toxicity with CAR-T therapy (20–22). However, whether development of CAR-T-related neurotoxicity increases the neurotoxicity of fundamental immunomodulators such as cyclosporine and tacrolimus after allo-HSCT is still unclear. In our study, four patients experienced Grade III neurotoxicity with seizures after CAR-T infusion. However, none of the four patients had developed drug-induced encephalopathy or TMA after transplant. Nevertheless, patients who present with severe neurotoxicity after CAR-T should be followed up and treated with caution.

There are now more and more published studies confirming that consolidative allo-HSCT following CAR-T therapy could reduce relapse rates and improve LFS for R/R ALL patients (6, 10, 11, 14–16). However, studies comparing CIR, LFS and OS between patients post CAR T-cell therapy and post-chemotherapy have not been reported. In the present study, we found similar CIR, LFS and OS rates between our two cohorts. Among our 105 patients, there was only 11% CIR, which is lower than the CIR rate reported previously among ALL patients in CR (33–35). There are multiple potential reasons for the relatively lower CIR in our study. First, a haploidentical donor was the main donor source (67%) in our study. Mo et al. found that a haplo-donor was superior to a matched sibling donor in offsetting the detrimental effects of high-risk factors and pre-transplant MSD among ALL patients (13, 36). Second, TBI-based conditioning regimes were used in the majority of our patients (83%). TBI has demonstrated an advantage over Bu as a component of conditioning regimens for MSD, MUD, and haplo-HSCT in pediatric and adult patients with ALL (33–35, 37). Third, myeloablative conditioning regimens were used in all the patients in this study, which were more effective in eradicating residual leukemia disease.

Several studies have previously shown the prognostic relevance of disease status and MRD status among B-ALL patients (38, 39). In our multivariate analysis, we demonstrated that the CIR for MRD+ patients before transplant was 3 times as high as that of MRD- patients and a negative MRD status either after CAR-T therapy or after chemotherapy and prior to transplant predicted better results. We showed that MRD before transplant was an independent predicator for CIR after HSCT and that achieving an MRD-negative CR was crucial and equally important for optimal transplantation outcomes among both the chemotherapy and CAR-T therapy groups.

Among patients receiving CAR-T therapy, whether CR patients with an CD19-negative status before transplant will have an increased risk of relapse remains a concern and will require further investigation. The possible reason for a CD19-negative relapse could be due to a selective immune escape mechanism of the tumor cells (40). So far there is no evidence that CD19-negative leukemic clones may be more easily attained as a further escape from the graft-versus-leukemia (GVL) effect of donor cells. Allo-HSCT is an anti-HLA immunotherapy, which is independent from CD19. Excluding the one early treatment-related death within one month following transplantation, half (2/4) of the patients relapsed with CD19-negative clones in our CAR-T group. Due to the limited number of cases in our study, we cannot make any conclusions on whether patients with a CD19-negative MRD status before transplant are more likely to relapse. Nevertheless, caution should be taken for those patients with CD19-negative MRD status who are to undergo an allo-HSCT.

In addition to CAR-T therapy, other immunotherapeutic approaches have proven successful in R/R B-ALL such as blimatumomab and inotuzomab. Currently, it remains unclear whether it is better to treat R/R B-ALL with CAR-T cell therapy *vs.*. blimatumomab or inotuzomab. Thus, it is important to conduct a randomized clinical trial in the future to investigate this question. A head-to-head comparison trial of blimatumomab or inotuzomab *vs.* CAR-T cell is undergoing (NCT03628053). Despite high CR/CRi of 67% achieved with inotuzomab for pediatric R/R ALL patients (41), 44% with blimatumomab (42), relapse remains the major problem. Without consolidative allo-HSCT, long-term disease control was limited with both blimatumomab and inotuzomab, especially for patients with high leukemic burden (43). The incidence of sinusoids obstruction syndrome (SOS), previously known as veno-occlusive disease, has been reported after allo-HSCT following inotuzomab, at an especially high rate (52%) in pediatric patients (41). In addition to efficacy and safety considerations, cost, insurance coverage, and local availability of each immunotherapy are all factors that will influence clinical decision-making. One limitation of our study is that it is not a randomized clinical trial but it is not feasible at the present time to do such randomized clinical trial as the CAR-T therapy is currently only indicated to chemotherapy refractory or relapsed patients. Nevertheless, our long-term follow-up and parallel comparison results demonstrate that pre-transplant CR induced by CAR-T therapy in R/R B-cell ALL patients carry the same prognostic significance as CR induced by conventional chemotherapy for patients without refractory disease. Although the CAR-T group had a higher incidence of Grade overall aGVHD and cGVHD, the incidence of serve aGVHD and cGVHD was comparable in both groups. Importantly, no clear increased transplant related mortality was identified in our CAR-T group. We conclude that the strategy of CAR-T therapy followed by allo-HSCT in R/R B cell-ALL was safe and effective, exhibiting similar long- term NRM, CIR, LFS and OS as those achieved among patients in the chemotherapy group.

## Data Availability Statement

The original contributions presented in the study are included in the article/supplementary material. Further inquiries can be directed to the corresponding author.

## Author Contributions

Y-LZ did the work of data collecting, data analysis and paper writing. D-PL and PL are the director of Lu Daopei Hospital and respond for the whole paper. Y-LZ, D-YL, R-JS, J-PZ, J-RZ, Z-JW, MX, X-YC, and YL take care of the all the HSCT patients. And J-fY and XZ took care of the patients during CAR-T therapy. All authors contributed to the article and approved the submitted version.

## Conflict of Interest

The authors declare that the research was conducted in the absence of any commercial or financial relationships that could be construed as a potential conflict of interest.
